# Quantifying *Cyanothece* growth under DIC limitation

**DOI:** 10.1016/j.csbj.2021.11.036

**Published:** 2021-11-29

**Authors:** Keisuke Inomura, Takako Masuda, Meri Eichner, Sophie Rabouille, Tomáš Zavřel, Jan Červený, Marie Vancová, Gábor Bernát, Gabrielle Armin, Pascal Claquin, Eva Kotabová, Susanne Stephan, David J. Suggett, Curtis Deutsch, Ondřej Prášil

**Affiliations:** a Graduate School of Oceanography, University of Rhode Island, Narragansett, Rhode Island, USA; b Institute of Microbiology, The Czech Academy of Sciences, Třeboň, Czech Republic; c Sorbonne Université, CNRS, Laboratoire d’Océanographie Microbienne, LOMIC, F-66650 Banyuls-sur-mer, France; d Department of Adaptive Biotechnologies, Global Change Research Institute, Czech Academy of Sciences, Brno, Czech Republic; e Laboratory of Electron Microscopy, Institute of Parasitology, Biology Centre of the Czech Academy of Sciences and Faculty of Science, University of South Bohemia, České Budějovice, Czech Republic; f Balaton Limnological Research Institute, Eötvös Loránd Research Network (ELKH), Tihany, Hungary; g Laboratoire de Biologie des ORganismes et Ecosystèmes Aquatiques (BOREA), UMR 8067, Muséum National d'Histoire Naturelle, CNRS, IRD Sorbonne Université, Université de Caen Normandie, Normandie Université, Esplanade de la Paix, F-14032 Caen, France; h Department Experimental Limnology, Leibniz Institute of Freshwater Ecology and Inland Fisheries, Stechlin, Germany; i University of Technology Sydney, Climate Change Cluster, Faculty of Science, Ultimo, NSW 2007, Australia; j School of Oceanography, University of Washington, Seattle, WA, USA

**Keywords:** *Cyanothece*, DIC, CO_2_, Nitrogen fixation, Nitrate, Diurnal cycle, Carbon, Carbon storage, Photosynthesis, Carbon allocation, Quantitative model, Mathematical model, Computer simulation, Cellular growth, Biomass, Growth limitation, Culture, Turbidostat

## Abstract

The photoautotrophic, unicellular N_2_-fixer, *Cyanothece,* is a model organism that has been widely used to study photosynthesis regulation, the structure of photosystems, and the temporal segregation of carbon (C) and nitrogen (N) fixation in light and dark phases of the diel cycle. Here, we present a simple quantitative model and experimental data that together, suggest external dissolved inorganic carbon (DIC) concentration as a major limiting factor for *Cyanothece* growth, due to its high C-storage requirement. Using experimental data from a parallel laboratory study as a basis, we show that after the onset of the light period, DIC was rapidly consumed by photosynthesis, leading to a sharp drop in the rate of photosynthesis and C accumulation. In N_2_-fixing cultures, high rates of photosynthesis in the morning enabled rapid conversion of DIC to intracellular C storage, hastening DIC consumption to levels that limited further uptake. The N_2_-fixing condition allows only a small fraction of fixed C for cellular growth since a large fraction was reserved in storage to fuel night-time N_2_ fixation. Our model provides a framework for resolving DIC limitation in aquatic ecosystem simulations, where DIC as a growth-limiting factor has rarely been considered, and importantly emphasizes the effect of intracellular C allocation on growth rate that varies depending on the growth environment.

## Graphical abstract

**Figure:**
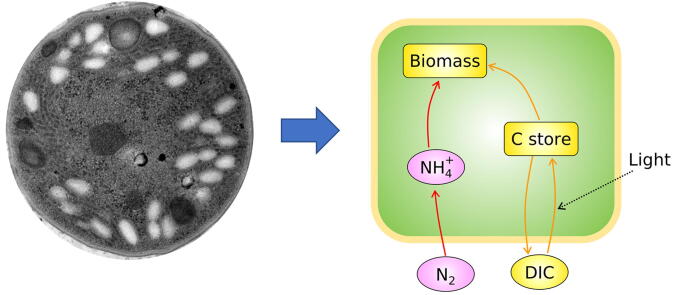

## 1 Introduction

By reducing atmospheric CO_2_ into bioavailable carbon (C), photosynthesis is the driving process of global ecosystem productivity and biogeochemical (nutrient) cycles. Phytoplanktonic organisms are responsible for most aquatic photosynthesis, and account for about half the primary production on earth [1]. A growing body of literature now reveals prokaryotic, nitrogen-fixing organisms as key players in the dynamics of phytoplanktonic communities and the world ocean's primary production. In particular, by their phototrophic capacity and their ability to fix molecular nitrogen (N_2_), unicellular N_2_-fixing cyanobacteria (UCYN) directly or indirectly contribute to and support primary production [2], [3], [4], exerting a direct coupling of the biogeochemical cycles of N and C [5], [6].

One of the most intensively studied organismal models of unicellular cyanobacteria is *Cyanothece* sp. ATCC 51142 (hereafter *Cyanothece*), which also has a capability to fix dinitrogen (N_2_) [7] to survive when bioavailable N, such as NH_4_^+^ or NO_3_^−^, is inaccessible. As in other photo-autotrophic, unicellular N_2_-fixing cyanobacteria (UCYN-B and -C), N_2_ fixation in *Cyanothece* is temporally segregated from carbon fixation [8], [9], [10], an evolution enabling protection of the O_2_-sensitive, nitrogenase enzyme responsible for N_2_ fixation [11]. Recent studies show that N_2_ fixation by UCYN-B is facilitated by the inactivation of PSII [12], [13], which may apply to *Cyanothece*. There are cases with in-complete temporal segregation depending on the light periodicity and cellular energy requirements, but the largest part of N_2_ fixation tends to occur at night [9], [14]. The temporal separation of photosynthesis and N_2_ fixation imposes these strains to rely on fixed carbon stored within cells as polysaccharides and on their subsequent respiration, which support the energy costs of N_2_ fixation. *Cyanothece* is not an obligate N_2_-fixer and grows well in the presence of bioavailable N, making it a relevant biological model of photo-autotrophic UCYN to investigate the cellular requirements imposed by N_2_ fixation on the cellular carbon metabolism, in comparison to nitrate-supported growth. The cellular growth of *Cyanothece* and its resulting population dynamics thus closely depend on the metabolic pathways of photosynthesis, respiration, NO_3_^−^ acquisition, and/or N_2_ fixation. Similar to other phytoplankton, the growth of autotrophic cyanobacteria is limited by the availability of macronutrients (nitrogen and phosphorus), trace metals (iron, copper) [15], [16], light, and temperature [17]. However, the effect of CO_2_ on their growth has only been started to be investigated intensively [10].

The effects of increasing CO_2_ on primary production are widely debated in the literature and motivated by the growing concern of ocean acidification [18], [19], [20], [21], [22]. Low DIC concentrations are likely to transiently occur [23] in areas with dense phytoplanktonic communities like the coastal regions, where *Cyanothece* are naturally present. Additionally, such low concentrations pose a potential, permanent risk in dense laboratory or industrial cultures and photo-bioreactors running without CO_2_ enrichment in the air supply. In the natural environment, we expect CO_2_ limitation to be altered following the increasing temperatures the world ocean is facing globally, but how dissolved inorganic carbon (DIC: the sum of CO_2_, HCO_3_^−^ and CO_3_^2−^) affects the growth of *Cyanothece* has not been analyzed in detail. Given the tight links between C and N metabolisms, what causes the growth difference between N_2_-fixing and NO_3_^−^ assimilating conditions under DIC limitation [10]?

**Fig. 1:**
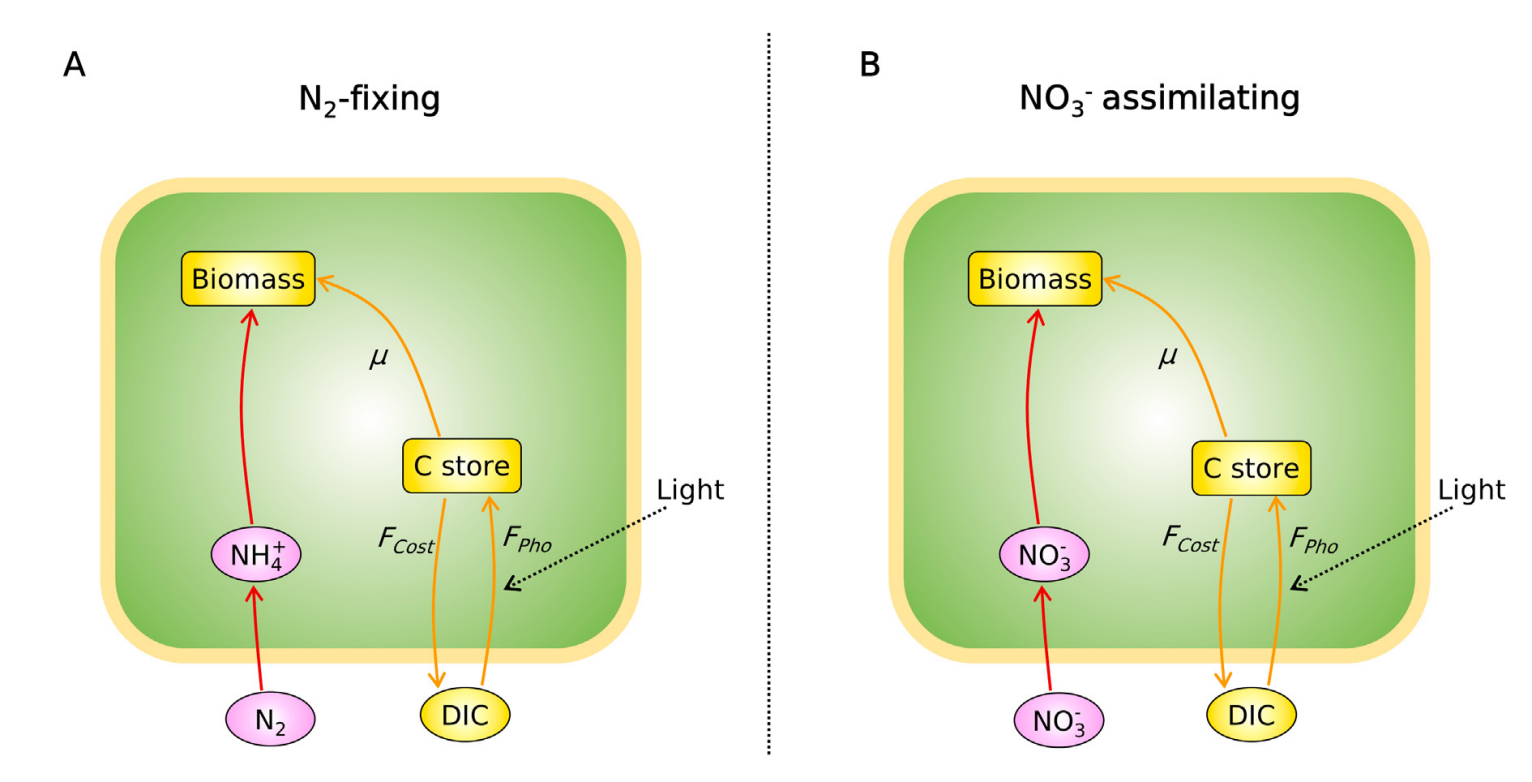
Schematics of the cell flux model of *Cyanothece* (CFM-Cyano) in (A) N_2_-fixing cells and in (B) NO_3_^−^ assimilating cells. The green boxes represent the cell. Ovals and rectangular boxes represent inorganic and organic molecules, respectively. Orange color represents C-dominant molecules and fluxes; while pink and red color represent N-dominant molecules and fluxes, respectively. *F_Pho_*, C fixation rate; *F_Cost_*, biosynthesis cost, which covers the electron and energy costs for biosynthesis, N_2_ fixation and NO_3_^−^ assimilation, thus differs between these two cases. See the definition in the main text below [eq. (7)].

Here, we implement a simple, yet mechanistic model of *Cyanothece* (Cell Flux Model of *Cyanothece*: CFM-Cyano) and quantitatively simulate the growth of this model organism, focusing on the control that DIC exerts on carbon fixation and on the subsequent C metabolism (Fig. 1: see Methods). This coarse-grained approach has an advantage in predicting concentrations of each metabolite pool [24], [25]. The flexibility and simplicity of CFM-Cyano allows the model to be adapted to different contexts (e.g., different datasets) and has provided intuitive overviews of cellular metabolism in unicellular N_2_-fixers [25], [26], [27], [28]. The present modeling work builds upon an experimental study of DIC limitation in the UCYN *Cyanothece* ATCC 51142 grown in turbidostats, both under a non-limiting nitrate supply and under obligate N_2_-fixation [10]. This experimental approach addressed the additional energetic burden that cells face when growing with N_2_ fixation compared to a NO_3_^−^-based growth. They also revealed how DIC limitation exerts a more severe control on N_2_-based growth compared with NO_3_^−^-supplied cultures. In this study, we provide a simple, mechanistic and quantitative representation of DIC limitation. Model results illustrate that resultant growth rates differ significantly between these metabolic modes, in relation to the intracellular allocation of fixed C.

## 2 Methods

### 2.1 Key equations

**Fig. 2:**
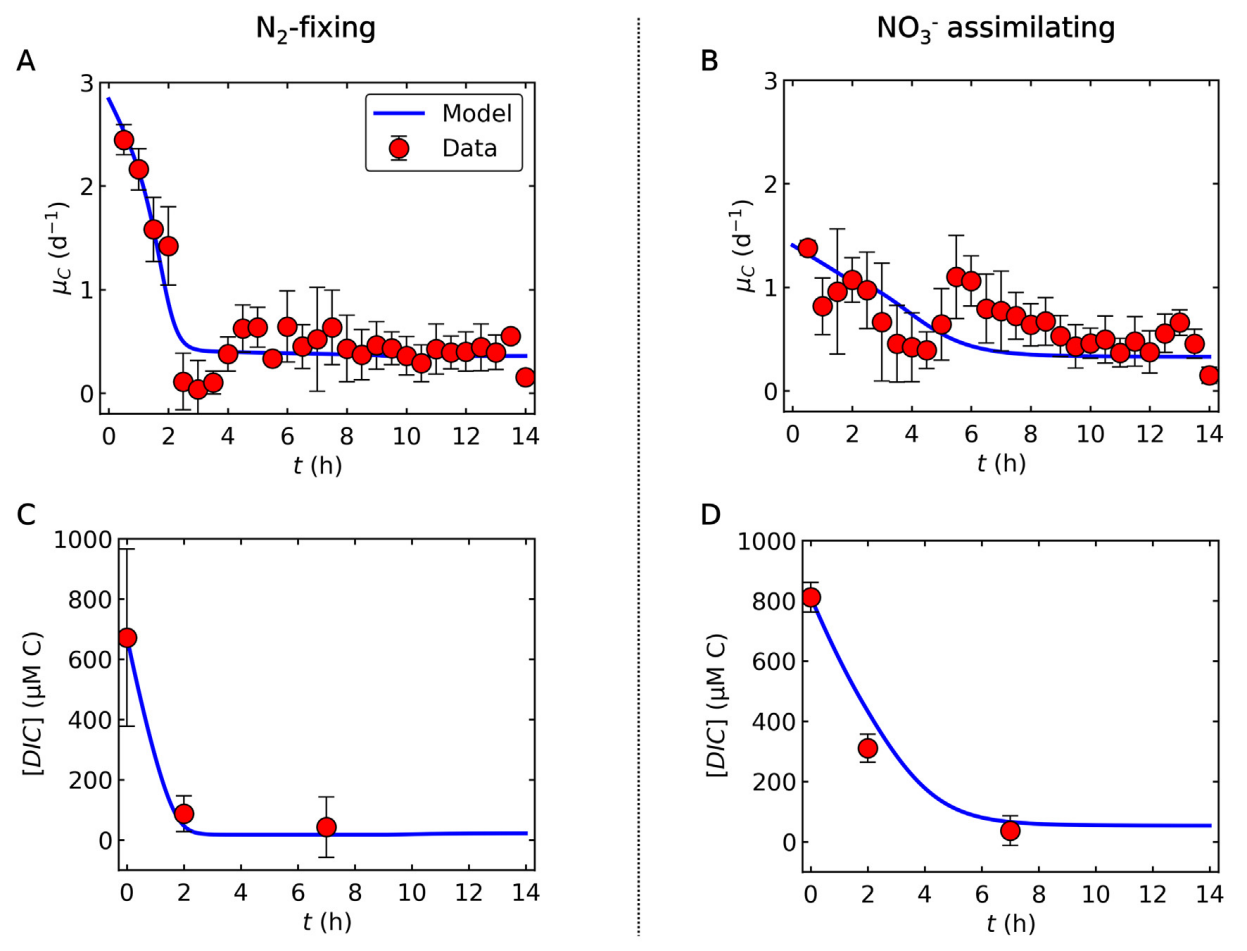
Relations between C-based growth rate and DIC (dissolved inorganic carbon) concentrations during the light period. (A) and (B) C-based growth rate *(μ_C_*) for N_2_-fixing and NO_3_^−^ assimilating cells, respectively. (C) and (D) DIC concentrations for N_2_-fixing and NO_3_^−^ assimilating cells, respectively. Blue curves are the results of model calculations, while red circles represent experimental data, deduced from growth rates determined by changes in OD_720_. Error bars represent standard deviation. The constancy of the DIC after h7 during the light period is supported by the observed constant pH [10].

**Fig. 3:**
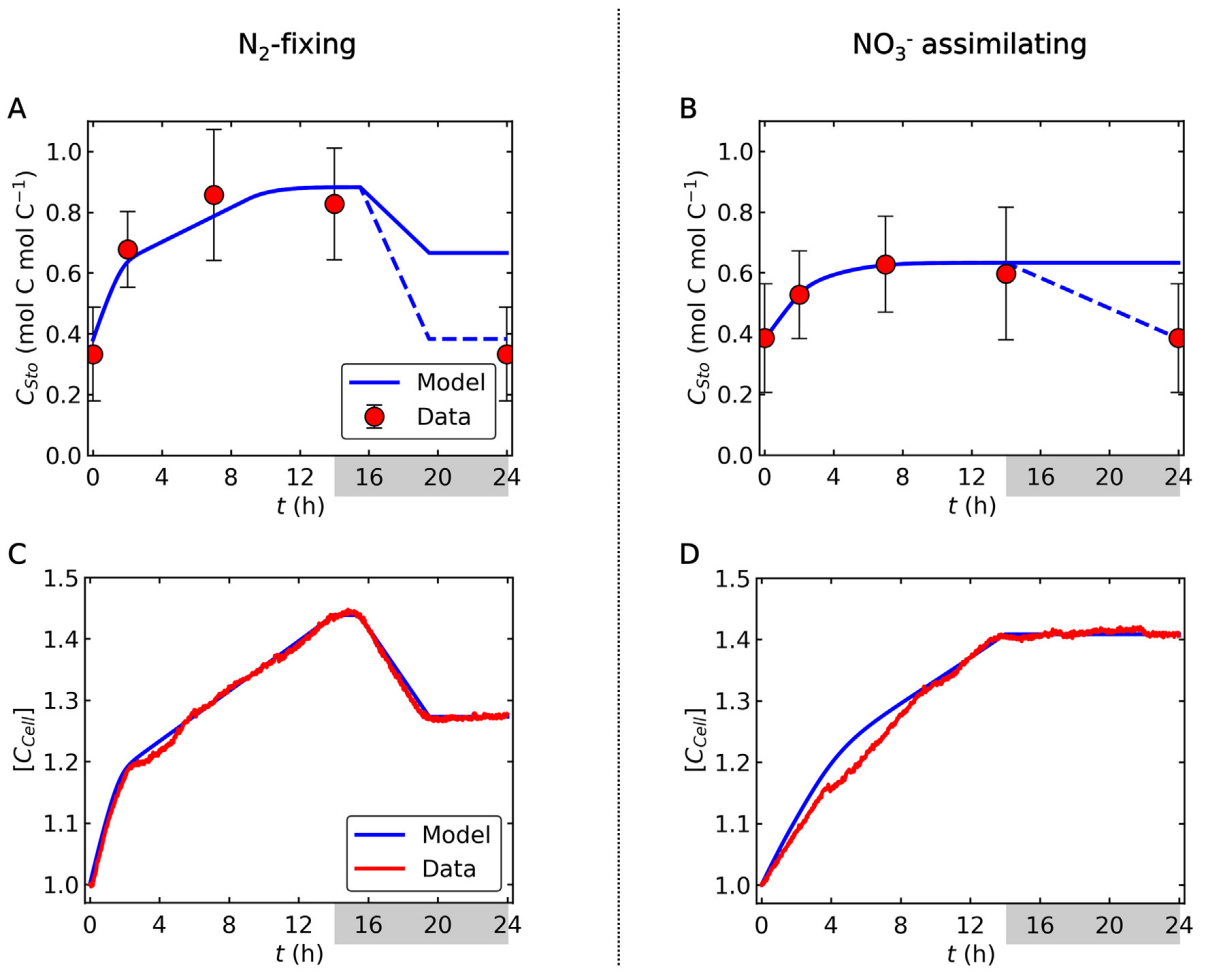
C storage and biomass C in N_2_-fixing and NO_3_^−^ assimilating cells. Blue curves are model results, while red circles and curves represent experimental data. The data for (A) and (B) are cellular polysaccharide content and those for (C) and (D) are OD_720_. The data of OD_720_ are shown as a relative value to the initial state. The sudden change in the slope at h14 represent the onset of the dark period. Also, N_2_ fixation is assumed between h14 and h20, which also causes the changes in the slope. In (A) and (B) error bars represent standard deviation and dashed lines shows the expected effect of C storage conversion to close the diurnal cycle.

The applied mechanistic model, CFM-Cyano, is based on a simplified metabolic flux network based on mass balances (Fig. 1) similar to previous CFMs [24], [29], [30] and earlier modeling on marine N_2_ fixers [31], [32], [33]. Most of these studies are reviewed in a recent publication [6]. CFM-Cyano simulated two metabolic scenarios: 1. N_2_-fixing (diazotrophic) and 2. NO_3_^−^ assimilating. Under the N_2_-fixing condition, N_2_ fixation accounted for the total N source, whereas under NO_3_^−^ assimilating condition, NO_3_^−^ was the total N source. Parameter units and values are listed in Supplementary Material (Table S1, S2). In the CFM-Cyano model, we considered C as the main “currency” of cellular growth, and computed the rates of photosynthesis, C storage production, and growth (biosynthesis) for each time step. The developed model was calibrated to reproduce the experimental data (Fig. 2, Fig. 3 and Fig. 6).

Cellular C is fixed by photosynthesis, whose rate depends on external DIC concentration, following Monod kinetics [34]:

(1) $$F_{\mathrm{Pho}}=F_{\mathrm{Pho}}^{\max}\left(\frac{\left[DIC\right]}{\left[DIC\right]+K_{\mathrm{DIC}}}\right)$$

where $F_{\mathrm{Pho}}$ is the rate of photosynthesis, $F_{\mathrm{Pho}}^{\max}$ is the maximum rate of photosynthesis, $\left[DIC\right]$ is DIC concentration in the culture, and $K_{\mathrm{DIC}}$ is the half saturation constant of DIC uptake. $F_{\mathrm{Pho}}$ was assumed zero during the night. While the intracellular CO_2_ concentration is the one that directly affects the rate of photosynthesis, the data for intracellular CO_2_ are not available and here we consider external DIC as a proxy for intracellular CO_2_. This implicitly assumes a linear relationship between internal and external pools of DIC. More complex relationships could arise from the presence of a carbon concentrating mechanism, and could be easily be incorporated in the model if substantiated by more direct evidence.

Once we determined the rate of photosynthesis, we then computed the net rate of C storage production, $F_{\mathrm{Csto}}$ , based on the difference between maximum C storage capacity, $C_{\mathrm{Sto}}^{\max}$ , and the current level of C storage, $C_{\mathrm{Sto}}$ , into starch-like molecules [35]:

(2) $$F_{\mathrm{Csto}}=min\left\{F_{\mathrm{Csto}}^{\max}\left(\frac{C_{\mathrm{Sto}}^{\max}-C_{\mathrm{Sto}}}{C_{\mathrm{Sto}}^{\max}}\right),F_{\mathrm{Pho}}\right\}$$

where the rate is proportional to $F_{\mathrm{Csto}}^{\max}$ , a maximum rate of C storage production. We adapted this formation from the Cell Flux Model of *Crocosphaera* (CFM-Croco) [30]. Since the storage production should not exceed the rate of photosynthesis, $F_{\mathrm{Csto}}$ was capped by $F_{\mathrm{Pho}}$ . Based on the mass balance, the rest of fixed C is used for growth. Thus, under N_2_ fixing case:

(3) $$\mu =\frac{F_{\mathrm{Pho}}-F_{\mathrm{Csto}}}{1+E}$$

where μ is the net growth rate, and *E* is a constant factor for respiration for providing energy for biosynthesis [25], [26], [29]. In reality, it is possible that stored C is used for the growth. Thus, the term $F_{\mathrm{Csto}}$ instead represented the net C storage production: the difference between gross C storage production and the loss for the growth. Under NO_3_^−^ assimilating case:

(4) $$\mu =\frac{F_{\mathrm{Pho}}-F_{\mathrm{Csto}}\left(1+E\right)}{1+E-C_{\mathrm{Sto}}E}$$

This formula counts the cost for NO_3_^−^ assimilation, to keep the cellular C:N constant as suggested by experimental data (see the section “*3.4. Cellular C:N and N assimilation*”). The derivations for [eq. (3)] and [eq. (4)] are in Supplementary text.

In this study, we simulated two types of *Cyanothece* cells: N_2_ fixing and non-N_2__-_fixing (Fig. 1). We provided different *E* values for the different N sources. Specifically, we followed the previously developed method, which computed *E* based on the mass, electron and energy balance [36]. Under NO_3_^−^ added case, NO_3_^−^ concentrations were abundant in the cultures (NO_3_^−^ culture; 16.16–22.67 mM), allowing us to focus on the C limitation. When NO_3_^−^ is not added, we assumed that there is sufficient N storage accumulated during the night to support biosynthesis. Since the data showed a decrease in biomass during the night, we allowed net cell growth only during the light periods (*μ* = 0 at night), although we were aware that cell division may occur also in the dark. We considered any excretion of carbohydrates as a part of carbon storage.

### 2.2 Time variations and model solutions

We then applied these four equations [eq. (1)]-[eq. (4)] to equations for the time variation in the experimental system of turbidostat cultures [10]. Here, the time variation of the non-C-stroage biomass concentration *X* increase based on the net growth rate [24]:

(5) $$\frac{\mathrm{dX}}{\mathrm{dt}}=\mu X$$

here, the loss term was not included since we compared the model results to the cumulative optical density. We use the following equation for the time dependence of cellular C storage per non-C-storage biomass $C_{\mathrm{Sto}}$ :

(6) $$\frac{dC_{\mathrm{Sto}}}{\mathrm{dt}}=F_{\mathrm{Csto}}-\mu C_{\mathrm{Sto}}-F_{\mathrm{Csto}}^{N2fix}$$

where $C_{\mathrm{Sto}}$ increases with C storage production, $F_{\mathrm{Csto}}$ , but decreases with cell growth ($\mu C_{\mathrm{Sto}}$), as $C_{\mathrm{Sto}}$ is converted to new cells during the light period. Also, during the dark period under N_2_-fixing conditions, $C_{\mathrm{Sto}}$ decreases with N_2_ fixation $F_{\mathrm{Csto}}^{N2fix}$ , which requires high consumption of C storage for intracellular O_2_ management and ATP generation [26], [29], [30], [33]. Under the NO_3_^−^ based condition, $F_{\mathrm{Csto}}^{N2fix}$ is zero. Finally, the time dependence of culture DIC is represented as follows:

(7) $$\frac{d\left[DIC\right]}{\mathrm{dt}}=F_{\mathrm{DIC}}^{\mathrm{Gas}}-k_{\mathrm{DIC}}^{\mathrm{Cell}}\left(F_{\mathrm{Pho}}-F_{\mathrm{Cost}}-F_{\mathrm{Csto}}^{N2fix}\right)$$

which is determined by the rate of gas exchange $F_{\mathrm{DIC}}^{\mathrm{Gas}}$ and the cellular DIC uptake (the second term). Here, $F_{\mathrm{DIC}}^{\mathrm{Gas}}$ , is proportional to the DIC disequilibrium with a rate coefficient $k_{\mathrm{DIC}}^{\mathrm{Gas}}$ : $F_{\mathrm{DIC}}^{\mathrm{Gas}}=\left({\left[DIC\right]}_{\mathrm{Eq}}-\left[DIC\right]\right)k_{\mathrm{DIC}}^{\mathrm{Gas}}$ . ${\left[DIC\right]}_{\mathrm{Eq}}$ is the equilibrium concentration of DIC in the environment, $k_{\mathrm{DIC}}^{\mathrm{Gas}}$ is the gas exchange constant, and $k_{\mathrm{DIC}}^{\mathrm{Cell}}$ is a constant factor for cellular DIC consumption, as a balance between photosynthesis, $F_{\mathrm{Pho}}$ , respiratory C cost, $F_{\mathrm{Cost}}$ (=μE for N_2_-based case, and $=E(F_{\mathrm{Csto}}-\mu C_{\mathrm{Sto}}+\mu )$ for NO_3_^−^-based case: see Supplementary text), and C consumption for N_2_ fixation during the dark period, $F_{\mathrm{Csto}}^{N2fix}$ .

We solved [eq. (5)]-[eq. (7)] with a finite difference method with $F_{\mathrm{Pho}}$ , $F_{\mathrm{Csto}}$ and *μ* computed for each time step from [eq. (1)]-[eq. (4)] with light:dark periods of 14 h:10 h, following the turbidostat experiment described in the companion paper [10]. We note that whereas a more detailed representation of C chemistry could be resolved [37], we chose to represent DIC as a pool for compatibility with the available data. Also, this way enabled us to keep our model simple with regard to extracellular carbonate chemistry and focus on a more detailed representation of intracellular carbon allocation over time. We assumed that influences of DIC speciation are relatively small compared to the large overall changes in DIC concentrations observed over the diel cycle.

Once we obtained the solutions for the time series, we computed cellular C content:

(8) $$\left[C_{\mathrm{Cell}}\right]=X\left(1+C_{\mathrm{Sto}}\right)$$

the relative value of which was compared with the values for optical density (OD_720_). We also computed the C-based growth rate $\mu_{C}$ :

(9) $$\mu_{C}=\frac{\mu +F_{\mathrm{Csto}}}{1+C_{\mathrm{Sto}}}$$

$\mu_{C}$ is formulated based on the net carbon assimilation rate normalized by the cellular C. $\mu_{C}$ was compared with the growth rate obtained from photobioreactor data, based on the change in the cumulative OD_720_ [10] (Fig. 3).

### 2.3 Obtaining N related values for N_2_ fixing case during the light period

During the light period under the N_2_-fixing condition, the rate of N_2_ fixation is small and the predicted integrated rate of biosynthesis is relatively small compared to that of C storage accumulation (Fig. 5). Thus, we approximate the cellular C:N, assuming a constant *N_Cell_*, the cellular N content per non-C-storage biomass C:

(10) $$C:N=\frac{1+C_{\mathrm{Sto}}}{N_{\mathrm{Cell}}}$$

### 2.4 Obtaining N related values for NO_3_^−^ added case during the light period

During the light period, the data showed largely constant cellular C:N (see below). Thus, we assumed constant cellular C:N. This allowed the computation of *N_Cell_* with the following equations:

(11) $$N_{\mathrm{Cell}}=\frac{1+C_{\mathrm{Sto}}}{C:N}$$

Also using C:N , assuming all the N source is NO_3_^−^, we could compute the NO_3_^−^ uptake rate *V_NO3_*:

(12) $$V_{NO3}=\frac{\mu_{C}}{C:N}$$

### 2.5 Laboratory measurements

We tested model solutions and constrained its unknown using time-dependent observations of the variation of intracellular C and N content, obtained during GAP 10th International meeting [10], [38]. Transmission electron microscopic (TEM) samples were processed as described in [38].

## 3 Results and discussion

### 3.1 C assimilation rate and DIC

The overall trend captures the data for both *μ_C_* (C assimilation rate) and DIC concentrations (Fig. 2). Under the N_2_-fixing condition, the model predicted a sharp decrease in *μ_C_* within ∼2 h (Fig. 2A), as DIC became depleted (Fig. 2C). In between these phases, experimental data showed a minimum, virtually zero growth after about 3 h in the light (h3), which was not captured by the model (Fig. 2A, B). This drop in *μ_C_* may indicate a lag phase [39], [40], [41] during which cells acclimate to a changed environment with low DIC by upregulating the activity of their CO_2_ concentration mechanisms, such as expression and synthesis of CO_2_ uptake systems and HCO_3_^−^ transporters [42], [43], [44], [45], [46], [47], [48]. This observation highlights that DIC may become a limiting factor for growth even when CO_2_ is supplied by air bubbling. In natural systems, severe DIC draw-down, comparable to our experimental set-up, may develop in freshwater systems with dense cyanobacterial blooms with predicted steady-state DIC concentrations of 130 to 230 µmol L^−1^ [37], in coastal regions [23], or within highly productive microenvironments such as cyanobacterial colonies in brackish water [49].

Under growth with NO_3_^−^, the initial growth rate was much lower than with N_2_-fixation. However, it remained relatively high after h2 until h6-h7 compared to N_2_-fixing culture (Fig. 2B). This concurred with a relatively high DIC level during this period (Fig. 2D). Experimental data for NO_3_^−^ assimilating cells also exhibited a significant drop in *μ_C_*, not seen in the model curve, likely due to the energy demand of acclimation (e.g., introduction of carbon concentration mechanism) as suggested above. The major difference between the two growth regimes (N_2_ *vs*. NO_3_^−^) is the initial rate of photosynthesis, which is highlighted by a higher $F_{\mathrm{Pho}}^{\max}$ for the N_2_-fixing condition. This difference can be explained by the energy and electron cost for NO_3_^−^ assimilation and intracellular C allocation (see *3.3. Fate of fixed C*).

### 3.2 Carbon storage and cellular C concentration

Model simulations of *C_Sto_* and [*C_Cell_*] (Fig. 3) were comparable to cellular polysaccharide levels and OD_720_, respectively, from cultures. The data-model consistency (Fig. 3) suggests that most of the C storage is in the form of polysaccharides, while OD_720_ is a proxy for total cellular C content rather than cell number. During the dark period under N_2_-fixing conditions, OD_720_ decreased drastically (Fig. 3C), reflecting the drop in polysaccharide content (Fig. 3A). At the beginning of the light period, *C_Sto_* increased rapidly but the increase was moderated as the rate of photosynthesis decreased due to DIC limitation (Fig. 2C, 3A). The cellular level of *C_Sto_* was higher for the N_2_-fixing condition than for the NO_3_^−^ supplementing treatment during the light period (Fig. 3A, B). However, the model predicts that *C_Sto_* in both treatments reaches the similar level at the end of the dark period due to the high C requirement for N_2_ fixation and O_2_ management.

Interestingly, whilst the model closely predicted the OD_720_ and the total biomass C concentration, at the end of the dark period, *C_Sto_* must return back to the initial value in the semi-steady state condition. This discrepancy may suggest that some of the C stored as polysaccharides is transformed to other molecules during the dark period. It is possible that a fraction of polysaccharides is used for synthesizing cyanophycin (N storing molecules with C:N of 2:1 [25]) or amino acids [38] or used to build structural elements such as the cell wall. In fact, protein synthesis from polysaccharides was observed during the night [38]. Such conversion must take place with negligible C consumption (i.e., small C storage loss to DIC) because the dark OD_720_ under NO_3_^−^ availability is almost constant (Fig. 3D); high C loss would have appeared as in the N_2_-fixing situation (Fig. 3C).

**Fig. 4:**
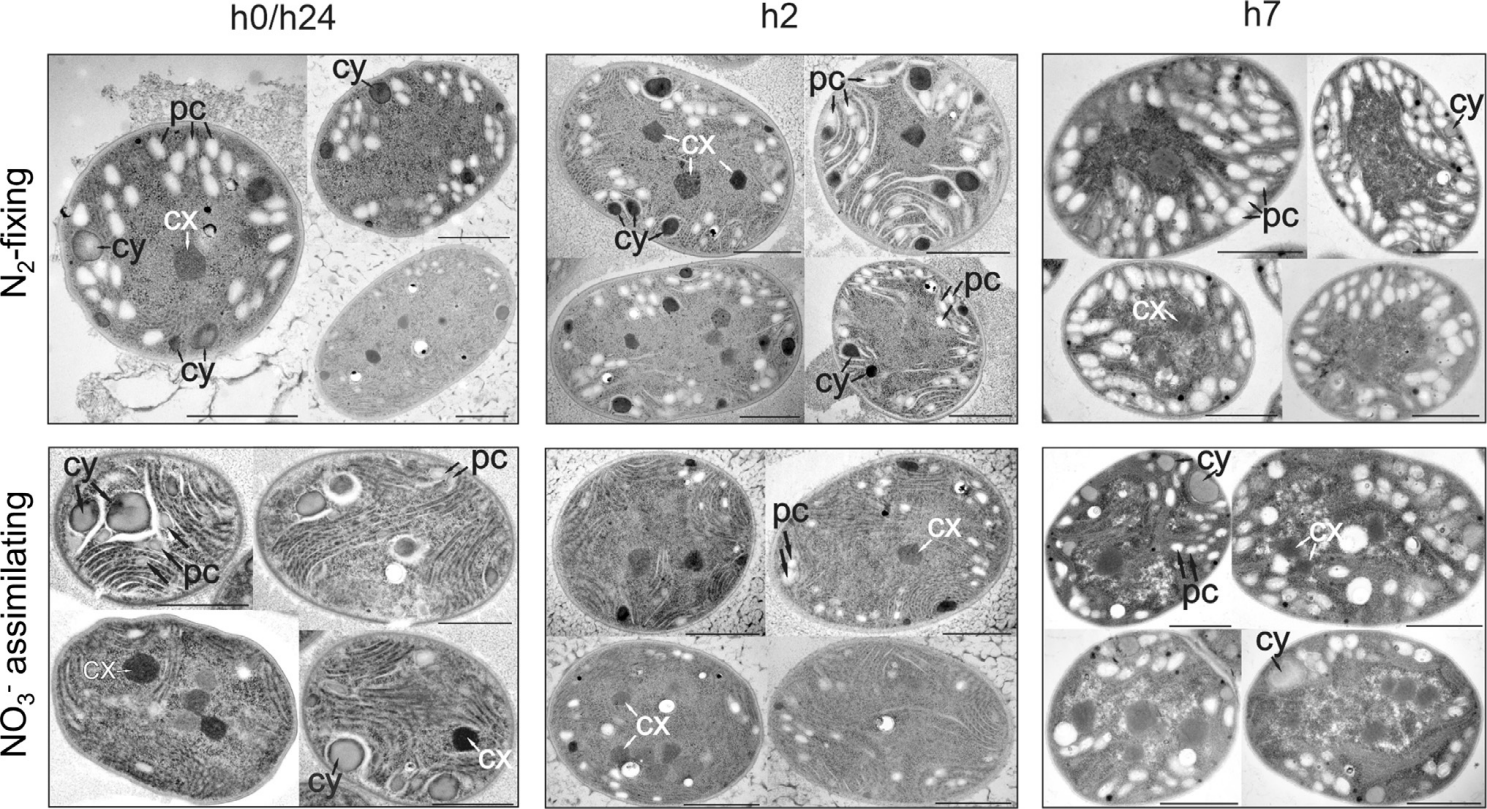
Transmission electron microscopic images of *Cyanothece* cells harvested at h0/h24, h2 and h7 in the light period. Top row – N_2_-fixing conditions; Bottom row – NO_3_^−^ assimilating conditions. pc; polysaccharide (C storage), cy; cyanophycin (N storage), and cx; carboxysome. Black bars show 1 µm. Additional images are available in Fig. S1.

Transmission electron microscopic (TEM) images taken at the beginning of the light period (thus, the end of the dark period) (Fig. 4, S1) showed more polysaccharide granules in N_2_-fixing cells than in the NO_3_^−^ grown ones, in contrast to bulk measurements of carbohydrate, OD_720,_ and the modelled *C_Sto_* (Fig. 3). This additional difference suggests that C, represented by *C_Sto_* and detected by the bulk analysis of carbohydrate content, includes C forms that are not visible as polysaccharide granules by TEM. The other forms of C could possibly be precursors of starches/carbohydrates of lower molecular weight [50]. Following this hypothesis, under NO_3_^−^-based conditions, more of the C would be present in this lower molecular weight form in the morning, potentially indicating a faster turnover of C under these conditions. Conversely, in the middle of the light phase (h7, Fig. 4, S1), TEM images show an increased number of polysaccharide granules in NO_3_^−^ assimilating cells, while bulk analysis of carbohydrate and modelled *C_Sto_* are higher in N_2_ fixing cells, indicating that degradation or turnover of carbon may be higher in N_2_ fixers at this time of day.

### 3.3 Fate of fixed C

**Fig. 5:**
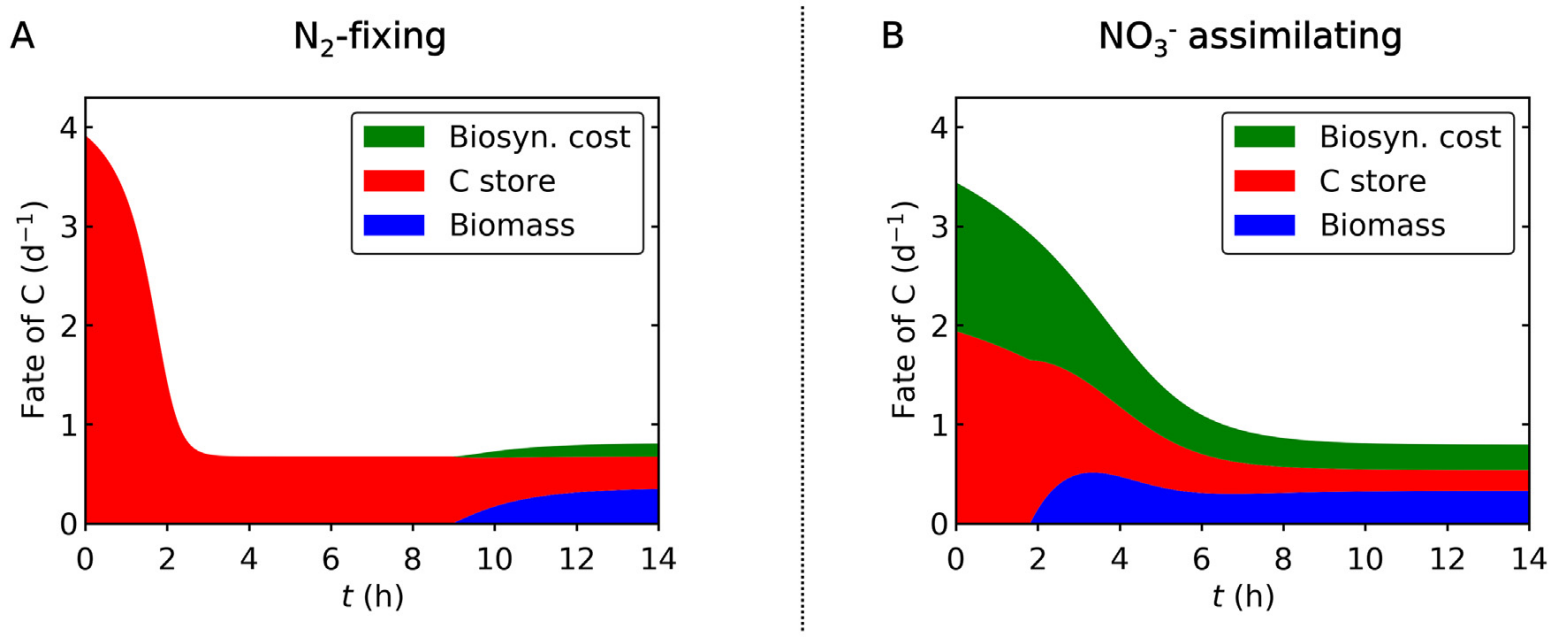
Fate of newly fixed C during the light period. (A) N_2_-fixing case. (B) NO_3_^−^ assimilating case. Green: biosynthesis cost. Red: C storage. Blue: C for non-C-storage biomass. Total value represents C fixation rates. The biosynthesis cost represents the sum of synthesis of non-C-storage biomass and the NO_3_^−^ assimilation.

The fate of fixed C is predicted to differ between the N_2_-fixing and NO_3_^−^ assimilating conditions (Fig. 5). Under N_2_-fixing condition, a significant fraction of C is initially channeled into C storage, leaving only a small fraction of newly fixed C for biosynthesis (cellular growth) (Fig. 5A). For non-N_2_-fixing cyanobacteria, it has been previously reported that biosynthesis is prioritized over C storage [38]. In contrast, our model suggests that N_2_-fixing unicellular cyanobacteria preferentially allocate fixed C to storage to support later N_2_ fixation through respiration at night. Indeed, during the early half of the light period, the model predicted that within the N_2_-fixing cells virtually all newly fixed C is accumulated in storage, while new C is allocated to biosynthesis only after the C storage reaches a certain threshold level at around h9 (Fig. 3A and Fig. 5A).

**Fig. 6:**
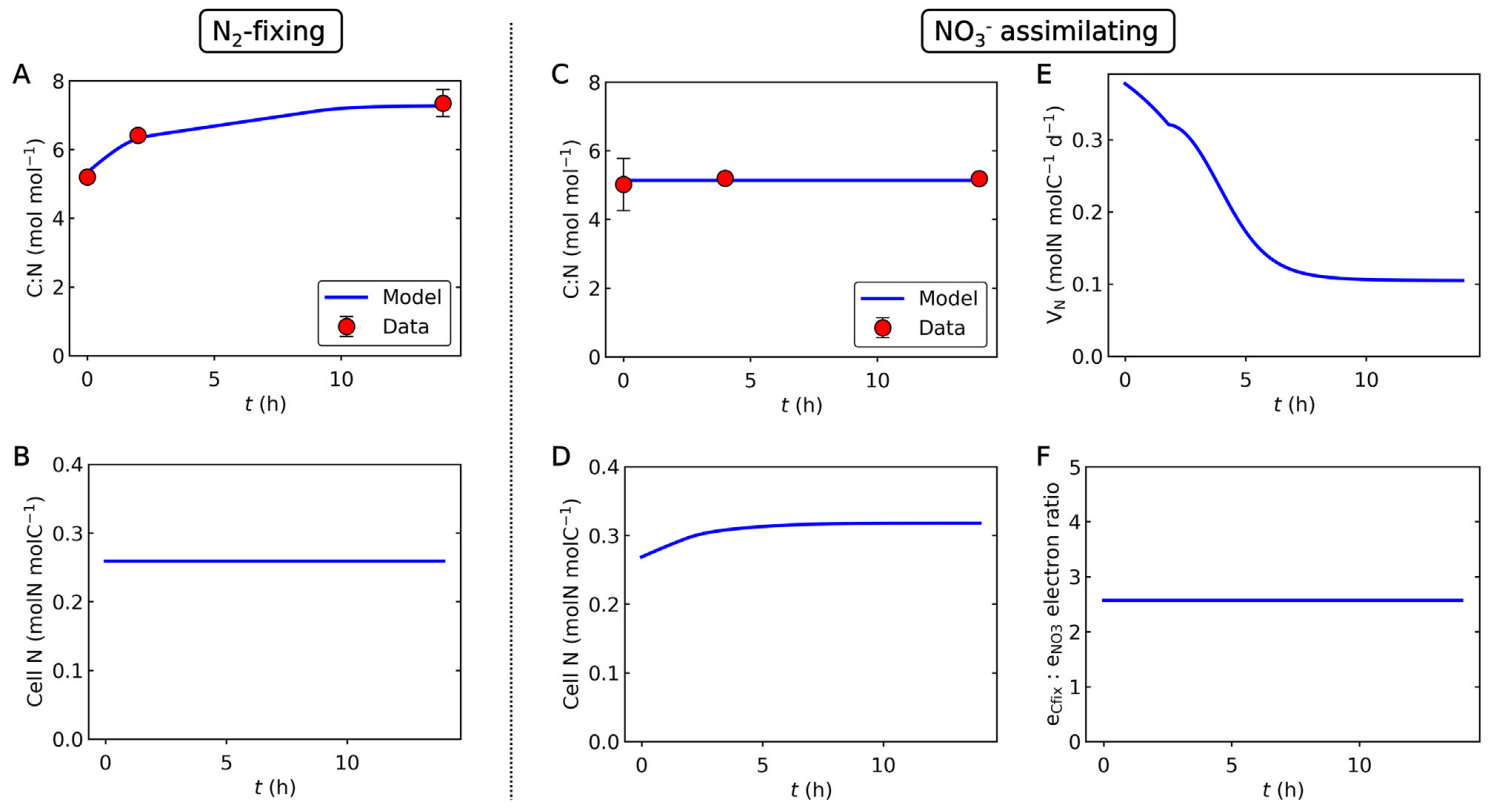
Cellular C:N ratio, N assimilation and electron allocation. (A) and (B) are under N_2_-fixing condition and (C) - (F) are under NO_3_^−^ added case. (A)(C) Cellular C:N. (B)(D) Cellular N per biomass C (which excludes C storage). (E) NO_3_^−^ uptake rate. (F) The ratio of electron used for C fixation to that for NO_3_^−^ reduction.

Contrary to the N_2_-fixing condition, when NO_3_^−^ is available, biosynthesis starts soon after the onset of the light period and continues up to the end of the light period (Fig. 5B). This occurs because the maximum level of *C_Sto_* is small and reaches its maximum much faster during the early light period (Fig. 3B), costing a significant amount of C. In the experiment, the total C fixation during the light period is similar between the two cases. However, given the high maximum rate of net C fixation under the N_2_-fixing conditions, if enough CO_2_ were continuously added to the culture to prevent DIC limitation, the rate of C fixation in the N_2_-fixing case might exceed the NO_3_^−^ assimilating case (Fig. S2). However, this simulation does not consider limitation by the availability of fixed N, which, in reality, would likely become limited under the N_2_-fixing case and constrain the rate of C fixation, since the N_2_ fixation occurs mainly during the night.

### 3.4 Cellular C:N ratios and N assimilation

Based on the modeled C metabolisms and C:N data, we have simulated cellular C:N and cellular N per biomass C (without C storage) for both the N_2_ fixing case and the NO_3_^−^ added case (Fig. 6). The data and the model revealed quantitative differences in daytime N metabolisms between these two cases. In the N_2_ fixing case, C:N of the cell increases (Fig. 6A) due to the accumulation of C storage (Fig. 3A). The cellular level of N is largely constant since N_2_ fixation does not occur (or is small) during the light period (Fig. 6B).

On the other hand, when NO_3_^−^ is added, the cellular C:N is largely constant (Fig. 6C) since the NO_3_^−^ uptake occurs simultaneously with the accumulation of C storage. Especially, during the early light period, the cellular N is enriched (Fig. 6D) due to NO_3_^−^ uptake (Fig. 6E). The model shows that the NO_3_^−^ uptake is about 200% larger during the early light period than the later light period, consistent with NanoSIMS results from the same experiment [38].

Based on the rate of NO_3_^−^ uptake and C fixation, we computed the ratio of electron use for these purposes (Fig. 6F). Despite the considerable rate of NO_3_^−^ uptake and high electron requirement for NO_3_^−^ reduction (8e^−^) relative to net C fixation (4e^−^) [36], the electron consumption for NO_3_^−^ is relatively small (∼1/2.57) (Fig. 6F). Thus, the use of electrons for NO_3_^−^ reduction is not sufficient to explain the difference in the rate of photosynthesis between the N_2_ fixing case and the NO_3_^−^ case during the light period, since the maximum rate of photosynthesis is about 100% higher for N_2_ fixing case (Fig. 2). The remaining difference can be explained by the energy cost (not electron cost) for NO_3_^−^ assimilation to biomass and the preferential allocation of C to C storage under the N_2_-fixing condition (Fig. 5).

### 3.5 DIC and C-storage requirements co-limit fate of fixed C

**Fig. 7:**
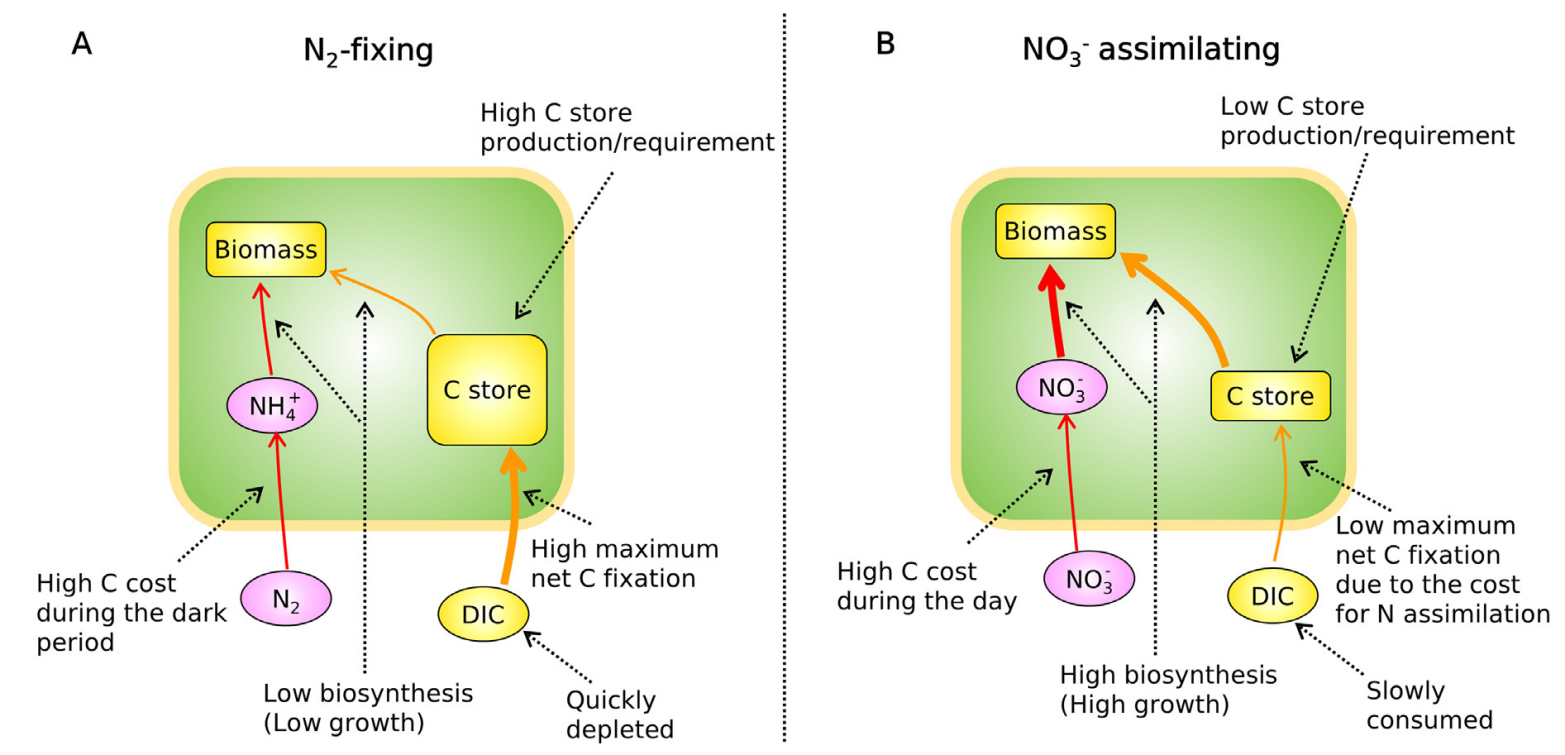
Summary of this study: differences in metabolisms between N_2_-fixing and NO_3_^−^ assimilating situations. (A) N_2_-fixing case. (B) NO_3_^−^ assimilating case. Under DIC limitation, N_2_-fixing cells have a lower growth rate despite the higher net maximum photosynthesis rate due to high C storage requirement.

Our model results highlight two major factors controlling cellular growth when the growth of *Cyanothece* is limited by inorganic C. Firstly, CO_2_ (DIC) availability limits the rate of photosynthesis, and then, the storage requirement limits the portion of newly fixed C that is used for biosynthesis or growth (Fig. 7). Under N_2_-fixing conditions, the maximum rate of C fixation ($F_{\mathrm{Pho}}^{\max}$) is higher. However, a large part of C is channeled into C storage, limiting the biosynthesis of new cells, thus limiting the growth rate. Secondly, despite the high maximum rate of photosynthesis in the N_2_-fixing condition, the photosynthesis rate rapidly decreases as it quickly depletes DIC. On the other hand, when NO_3_^−^ is available, a large part of fixed C is channeled directly into biosynthesis, thus resulting in higher growth (Fig. 7). The lower maximum rate of photosynthesis works favorably under DIC limitation since it keeps ambient DIC relatively high. However, if limitation by DIC becomes less severe, due to the high photosynthetic capacity, the cells under N_2_-fixing conditions might grow even faster, yielding a potential co-limitation of DIC and fixed N. This hypothesis needs to be tested with further experiments.

## 4 Conclusions

We have developed a simple, cellular model of *Cyanothece* (CFM-Cyano) focusing on DIC limitation. The model reproduced laboratory data both for N_2_-fixing and NO_3_^−^ available conditions demonstrating that, under N_2_-fixing conditions, C storage is prioritized during the early photoperiod to accumulate C in storage for N_2_ fixation during the night, and later during the day, biosynthesis increases. This two-step growth limitation may apply to other photoautotrophic unicellular N_2_-fixers, such as *Crocosphaera watsonii*.

A recent study pinpointed the risk of significant biases brought by a lack of control of the DIC supply in cultures of *Cyanothece* [10]. Our study further emphasizes the potential for DIC limitation in laboratory studies, which may severely limit the growth rate of any photoautotrophs and may have been overlooked as a critical regulatory factor in previous studies. Our model is simple and efficient and can be incorporated into sophisticated ecological or physiological models to resolve intracellular carbon allocation, especially under conditions when DIC availability becomes limiting, such as dense cyanobacterial blooms or biotechnological mass cultures.

## 5 Model availability

CFM-Cyano is freely available from Zenodo at https://zenodo.org/record/3740246 (DOI: 10.5281/zenodo.3740245).

## Author contributions

KI developed and run the model with suggestions from TM, ME, SR and OP. KI, TM and OP administered the project. TM, ME, SR, TZ, JČ, MV, GB, PC, EK, SS, DJS, OP contributed to obtaining data. KI wrote the original manuscript, which is revised by KI, TM, ME, SR, TZ, MV, GB, GA, PC, EK, SS, DJS, CD, OP.

## Declaration of Competing Interest

The authors declare that they have no known competing financial interests or personal relationships that could have appeared to influence the work reported in this paper.

## Footnotes

Appendix A Supplementary data to this article can be found online at https://doi.org/10.1016/j.csbj.2021.11.036.

## References

[1] Field C.B., Behrenfeld M.J., Randerson J.T., Falkowski P. (1998). Primary production of the biosphere: Integrating terrestrial and oceanic components. Science.

[2] Zehr J.P., Waterbury J.B., Turner P.J., Montoya J.P., Omoregie E., Steward G.F. (2001). Unicellular cyanobacteria fix N_2_ in the subtropical North Pacific Ocean. Nature.

[3] Montoya J.P., Holl C.M., Zehr J.P., Hansen A., Villareal T.A., Capone D.G. (2004). High rates of N_2_ fixation by unicellular diazotrophs in the oligotrophic Pacific Ocean. Nature.

[4] Moisander P.H., Beinart R.A., Hewson I., White A.E., Johnson K.S., Carlson C.A. (2010). Unicellular cyanobacterial distributions broaden the oceanic N_2_ fixation domain. Science.

[5] Gruber N., Galloway J.N. (2008). An Earth-system perspective of the global nitrogen cycle. Nature.

[6] Inomura K., Deutsch C., Masuda T., Prášil O., Follows M.J. (2020). Quantitative models of nitrogen-fixing organisms. Comput Struct Biotechnol J.

[7] Reddy K.J., Haskell J.B., Sherman D.M., Sherman L.A. (1993). Unicellular, aerobic nitrogen-fixing cyanobacteria of the genus *Cyanothece*. J Bacteriol.

[8] Meunier P.C., Colón-lópez M.S., Sherman L.A. (1997). Temporal changes in state transitions and photosystem organization in the unicellular, diazotrophic cyanobacterium Cyanothece sp. ATCC 5112. Plant Physiol.

[9] Rabouille S., Van de Waal D.B., Matthijs H.C.P., Huisman J. (2014). Nitrogen fixation and respiratory electron transport in the cyanobacterium Cyanothece under different light/dark cycles. FEMS Microbiol Ecol.

[10] Rabouille S., Campbell D.A., Masuda T., Zavřel T., Bernat G., Polerecky L. (2021). Electron and biomass dynamics of *Cyanothece* under interacting nitrogen and carbon limitations. Front Microbiol.

[11] Gallon J.R. (1992). Tansley Review No. 44 Reconciling the incompatible: N_2_ fixation and O_2_. New Phytol.

[12] Rabouille S., Claquin P. (2016). Photosystem-II shutdown evolved with nitrogen fixation in the unicellular diazotroph Crocosphaera watsonii. Environ Microbiol.

[13] Masuda T., Bernát G., Bečková M., Kotabová E., Lawrenz E., Lukeš M. (2018). Diel regulation of photosynthetic activity in the oceanic unicellular diazotrophic cyanobacterium *Crocosphaera watsonii* WH8501. Environ Microbiol.

[14] Dron A., Rabouille S., Claquin P., Talec A., Raimbault V., Sciandra A. (2013). Photoperiod length paces the temporal orchestration of cell cycle and carbon-nitrogen metabolism in Crocosphaera watsonii. Environ Microbiol.

[15] Moore C.M., Mills M.M., Arrigo K.R., Berman-Frank I., Bopp L., Boyd P.W. (2013). Processes and patterns of oceanic nutrient limitation. Nat Geosci.

[16] Huertas M., López-Maury L., Giner-Lamia J., Sánchez-Riego A., Florencio F. (2014). Metals in cyanobacteria: Analysis of the copper, nickel, cobalt and arsenic homeostasis mechanisms. Life.

[17] Dechatiwongse P., Srisamai S., Maitland G., Hellgardt K. (2014). Effects of light and temperature on the photoautotrophic growth and photoinhibition of nitrogen-fixing cyanobacterium *Cyanothece* sp. ATCC 51142. Algal Res.

[18] Riebesell U., Wolf-Gladrow D.A., Smetacek V. (1993). Carbon dioxide limitation of marine phytoplankton growth rates. Nature.

[19] Gattuso J.P., Magnan A., Billé R., Cheung W.W.L., Howes E.L., Joos F. (2015). Contrasting futures for ocean and society from different anthropogenic CO_2_ emissions scenarios. Science.

[20] Yang Y., Hansson L., Gattuso J.-P. (2016). Data compilation on the biological response to ocean acidification: An update. Earth Syst Sci Data.

[21] Gao K., Beardall J., Häder D.P., Hall-Spencer J.M., Gao G., Hutchins D.A. (2019). Effects of ocean acidification on marine photosynthetic organisms under the concurrent influences of warming, UV radiation, and deoxygenation. Front Mar Sci.

[22] Eichner M., Rost B., Kranz S.A. (2014). Diversity of ocean acidification effects on marine N_2_ fixers. J Exp Mar Biol Ecol.

[23] Evans W., Hales B., Strutton P.G. (2011). Seasonal cycle of surface ocean pCO_2_ on the Oregon shelf. J Geophys Res Oceans.

[24] Inomura K., Masuda T., Gauglitz J.M. (2019). Active nitrogen fixation by *Crocosphaera* expands their niche despite the presence of ammonium – A case study. Sci Rep.

[25] Inomura K., Omta A.W., Talmy D., Bragg J., Deutsch C., Follows M.J. (2020). A Mechanistic model of macromolecular allocation, elemental stoichiometry, and growth rate in phytoplankton. Front Microbiol.

[26] Inomura K., Bragg J., Riemann L., Follows M.J., Virolle M.-J. (2018). A quantitative model of nitrogen fixation in the presence of ammonium. PLoS One.

[27] Inomura K., Wilson S.T., Deutsch C., Gilbert J. (2019). Mechanistic model for the coexistence of nitrogen fixation and photosynthesis in marine *Trichodesmium*. mSystems.

[28] Inomura K., Follett C.L., Masuda T., Eichner M., Prášil O., Deutsch C. (2020). Carbon transfer from the host diatom enables fast growth and high rate of N_2_ fixation by symbiotic heterocystous cyanobacteria. Plants.

[29] Inomura K., Bragg J., Follows M.J. (2017). A quantitative analysis of the direct and indirect costs of nitrogen fixation: a model based on *Azotobacter vinelandii*. ISME J.

[30] Inomura K., Deutsch C., Wilson S.T., Masuda T., Lawrenz E., Bučinská L. (2019). Quantifying oxygen management and temperature and light dependencies of nitrogen fixation by Crocosphaera watsonii. mSphere.

[31] Rabouille S., Staal M., Stal L.J., Soetaert K. (2006). Modeling the dynamic regulation of nitrogen fixation in the cyanobacterium *Trichodesmium* sp. Appl Environ Microbiol.

[32] Agawin N.S.R., Rabouille S., Veldhuis M.J.W., Servatius L., Hol S., van Overzee H.M.J. (2007). Competition and facilitation between unicellular nitrogen-fixing cyanobacteria and non-nitrogen-fixing phytoplankton species. Limnol Oceanogr.

[33] Grimaud G.M., Rabouille S., Dron A., Sciandra A., Bernard O. (2014). Modelling the dynamics of carbon – nitrogen metabolism in the unicellular diazotrophic cyanobacterium *Crocosphaera watsonii* WH8501, under variable light regimes. Ecol Model.

[34] Monod J. (1949). The growth of bacterial cultures. Ann Rev Mar Sci.

[35] Deschamps P., Colleoni C., Nakamura Y., Suzuki E., Putaux J.-L., Buleon A. (2008). Metabolic symbiosis and the birth of the plant kingdom. Mol Biol Evol.

[36] Rittmann B.E., McCarty P.L. (2001).

[37] Ji X., Verspagen J.M.H., van de Waal D.B., Rost B., Huisman J. (2020). Phenotypic plasticity of carbon fixation stimulates cyanobacterial blooms at elevated CO_2_. Sci Adv.

[38] Polerecky L., Masuda T., Eichner M., Rabouille S., Vancová M., Kienhuis M.V.M. (2021). Temporal patterns and intra- and inter-cellular variability in carbon and nitrogen assimilation by the unicellular cyanobacterium *Cyanothece* sp. ATCC 51142. Front Microbiol.

[39] Swinnen I.A.M., Bernaerts K., Dens E.J.J., Geeraerd A.H., Van Impe J.F. (2004). Predictive modelling of the microbial lag phase: A review. Int J Food Microbiol.

[40] Mulderij G., Mooij W.M., Smolders A.J.P., Donk E.V. (2005). Allelopathic inhibition of phytoplankton by exudates from Stratiotes aloides. Aquat Bot.

[41] Rolfe M.D., Rice C.J., Lucchini S., Pin C., Thompson A., Cameron A.D.S. (2012). Lag phase is a distinct growth phase that prepares bacteria for exponential growth and involves transient metal accumulation. J Bacteriol.

[42] Miller A.G., Colman B. (1980). Active transport and accumulation of bicarbonate by a unicellular cyanobacterium. J Bacteriol.

[43] Miller A.G., Espie G.S., Canvin D.T. (1990). Physiological aspects of CO_2_ and HCO_3_^−^ transport by cyanobacteria: a review. Can J Bot.

[44] Kaplan A., Badger M.R., Berry J.A. (1980). Photosynthesis and the intracellular inorganic carbon pool in the bluegreen alga *Anabaena variabilis*: Response to external CO_2_ concentration. Planta.

[45] Badger MR, Spalding MH (2000) CO_2_ acquisition, concentration and fixation in cyanobacteria and algae. In: Leegood RC, Sharkey TD and von Caemmerer S (eds), Advances in Photosynthesis, Vol 9. Photosynthesis: Physiology and Metabolism. 9: 399–434.

[46] Price G.D., Badger M.R., Woodger F.J., Long B.M. (2008). Advances in understanding the cyanobacterial CO_2_-concentrating-mechanism (CCM): Functional components, Ci transporters, diversity, genetic regulation and prospects for engineering into plants. J Exp Bot.

[47] Ogawa T., Kaplan A. (2003). Inorganic carbon acquisition systems in cyanobacteria. Photosynth Res.

[48] Eichner M., Thoms S., Kranz S.A., Rost B. (2015). Cellular inorganic carbon fluxes in Trichodesmium: A combined approach using measurements and modelling. J Exp Bot.

[49] Ploug H., Adam B., Musat N., Kalvelage T., Lavik G., Wolf-Gladrow D. (2011). Carbon, nitrogen and O_2_ fluxes associated with the cyanobacterium Nodularia spumigena in the Baltic Sea. ISME J.

[50] Zavřel T., Očenášová P., Sinetova M., Červený J. (2018). Determination of Storage (Starch/Glycogen) and Total Saccharides Content in Algae and Cyanobacteria by a Phenol-Sulfuric Acid Method. Bio-Protocol..

