# Supplementary material for: Quantifying Cyanothece growth under DIC limitation
Source: Comput Struct Biotechnol J. 2021 Nov 29;19:6456–64. doi: 10.1016/j.csbj.2021.11.036 (PMC8665340; doi:10.1016/j.csbj.2021.11.036)
Supplement: Supplementary data 1 [file mmc1.pdf]

Supplementary text

To find the growth rate under the N_2 fixing case, we use eq. 3 from the manuscript and derive it from the equation below.

$$F_{Csto} = F_{Pho} - \mu(1 + E) \qquad \text{[eq. S1]}$$

F_{Csto} represents the net carbon storage production. The carbon fixation rate, F_{Pho}, adds to carbon storage, whereas the second term represents a loss. μE is the respiration rate associated with biomass production and μ represents the net growth rate, so the entire loss term represents the biomass production rate. This term is signified as a loss term because biomass production requires carbon from storage to grow. Solving eq. S1 for μ gives eq. 3 as follows:

$$F_{Csto} + \mu(1 + E) = F_{Pho}$$

$$\mu(1 + E) = F_{Pho} - F_{Csto}$$

$$\frac{\mu(1 + E)}{(1 + E)} = \frac{F_{Pho} - F_{Csto}}{(1 + E)}$$

$$\mu = \frac{F_{Pho} - F_{Csto}}{(1 + E)} \qquad \text{[eq. 3]}$$

To find the growth rate under the NO_3^- assimilating case, we use eq.4 from the manuscript and derive it from the equation below.

$$F_{Pho} = F_{Csto} + E(F_{Csto} - \mu C_{sto}) + \mu(1 + E) \qquad \text{[eq. S2]}$$

Again, F_{Pho} represents the rate of carbon fixation, F_{Csto} is the net carbon storage productions, and $\mu(1 + E)$ is the biomass production rate. The middle term, $E(F_{Csto} - \mu C_{sto})$, indicates the cost for nitrogen enrichment in the cell. $F_{Csto} - \mu C_{sto}$ tells how much carbon is increased in the cell during carbon fixation. In laboratory experiments, the C:N remains constant, so nitrogen enrichment must be proportional to carbon enrichment. Therefore, the $F_{Csto} - \mu C_{sto}$ can be expressed in terms of nitrogen by applying E, which represents a cost for nitrogen assimilation per C. Solving eq. S1 for μ gives eq. 4 as follows:

$$F_{Pho} = F_{Csto} + EF_{Csto} - E\mu C_{sto} + \mu + \mu E$$

$$F_{Pho} - F_{Csto} - EF_{Csto} = -E\mu C_{sto} + \mu + \mu E$$

$$F_{Pho} - F_{Csto} - EF_{Csto} = -E\mu C_{sto} + \mu + \mu E$$

$$F_{Pho} - F_{Csto}(1 + E) = \mu(1 + E - EC_{sto})$$

$$\frac{F_{Pho} - F_{Csto}(1 + E)}{(1 + E - EC_{sto})} = \frac{\mu(1 + E - EC_{sto})}{(1 + E - EC_{sto})}$$

$$\mu = \frac{F_{Pho} - F_{Csto}(1+E)}{1 + E - EC_{sto}} \qquad \text{[eq. 4]}$$

Respiratory costs

Respiratory costs (F_{Cost}) are represented by the term with E. Thus, under diazotrophic condition, from eq. S1, $F_{Cost} = \mu E$. Similarly, under NO_3^- assimilating condition, $F_{Cost} = E(F_{Csto} - \mu C_{sto}) + \mu E = E(F_{Csto} - \mu C_{Sto} + \mu)$.
