# Supplementary material for: Quantifying Cyanothece growth under DIC limitation
Source: Comput Struct Biotechnol J. 2021 Nov 29;19:6456–64. doi: 10.1016/j.csbj.2021.11.036 (PMC8665340; doi:10.1016/j.csbj.2021.11.036)
Supplement: Supplementary data 2 [file mmc2.pdf]

Table S1 Parameters, units and definitions

Parameter	Unit	Definition
F_{Pho}	d^{-1}	Photosynthesis rate
F_{Pho}^{max}	d^{-1}	Maximum photosynthesis rate
$[DIC]$	μmol l^{-1}	DIC concentration in the environment
K_{DIC}	μmol l^{-1}	Half saturation constant for DIC
F_{Csto}	d^{-1}	C storage production
F_{Csto}^{max}	d^{-1}	Maximum C storage production
C_{Sto}^{max}	dimensionless	Maximum C storage per biomass
C_{Sto}	dimensionless	C storage per biomass
μ	d^{-1}	Net growth rate
E	dimensionless	Respiration factor
X	μmol l^{-1}	Cumulative biomass concentration
t	d	Time
F_{Csto}^{N2fix}	d^{-1}	C consumption for N_2 fixation
F_{DIC}^{Gas}	μmol l^{-1} d^{-1}	Gas exchange rate of DIC
$[DIC]_{Eq}$	μmol l^{-1}	Equilibrium DIC concentration
k_{DIC}^{Gas}	d^{-1}	Gas exchange constant for DIC
k_{DIC}^{Cell}	μmol l^{-1}	DIC uptake factor
F_{Cost}	d^{-1}	Respiratory C cost
$[C_{Cell}]$	μmol l^{-1}	Cellular C concentration
μ_C	d^{-1}	C-based growth rate
$C:N$	dimensionless	Cellular C:N ratio
N_{Cell}	dimensionless	Cellular N content per biomass C

Table S2 Parameter values

Parameter	Value	Unit
F_{Pho}^{max}	4.5	d^{-1}
F_{Csto}^{max}	8	d^{-1}
$[DIC]_{Eq}$	1000	μmol l^{-1}
k_{DIC}^{Cell}	2.9×10^3	μmol l^{-1}
Diazotrophic case		
K_{DIC}	100	μmol l^{-1}
C_{Sto}^{max}	0.92	dimensionless
E	0.383*1	dimensionless
F_{Csto}^{N2fix}	1.3	d^{-1}
k_{DIC}^{Gas}	2	d^{-1}
N_{Cell}	0.259	dimensionless
NO_3^- added case		
K_{DIC}	250	μmol l^{-1}
C_{Sto}^{max}	0.65	dimensionless
E	0.774*2	dimensionless
F_{Csto}^{N2fix}	0	d^{-1}
k_{DIC}^{Gas}	1.65	d^{-1}
$C:N$	5.14	dimensionless

*1,2 Value based on [1] with NH^{4+} and NO^{3-} for N substrate, respectively.

Reference

1. Rittmann BE, McCarty PL (2001.) Environmental Biotechnology: Principles and Applications. McGraw-Hill: New York, NY.
