# Supplementary material for: Quantifying Cyanothece growth under DIC limitation
Source: Comput Struct Biotechnol J. 2021 Nov 29;19:6456–64. doi: 10.1016/j.csbj.2021.11.036 (PMC8665340; doi:10.1016/j.csbj.2021.11.036)
Supplement: Supplementary data 3 [file mmc3.pdf]

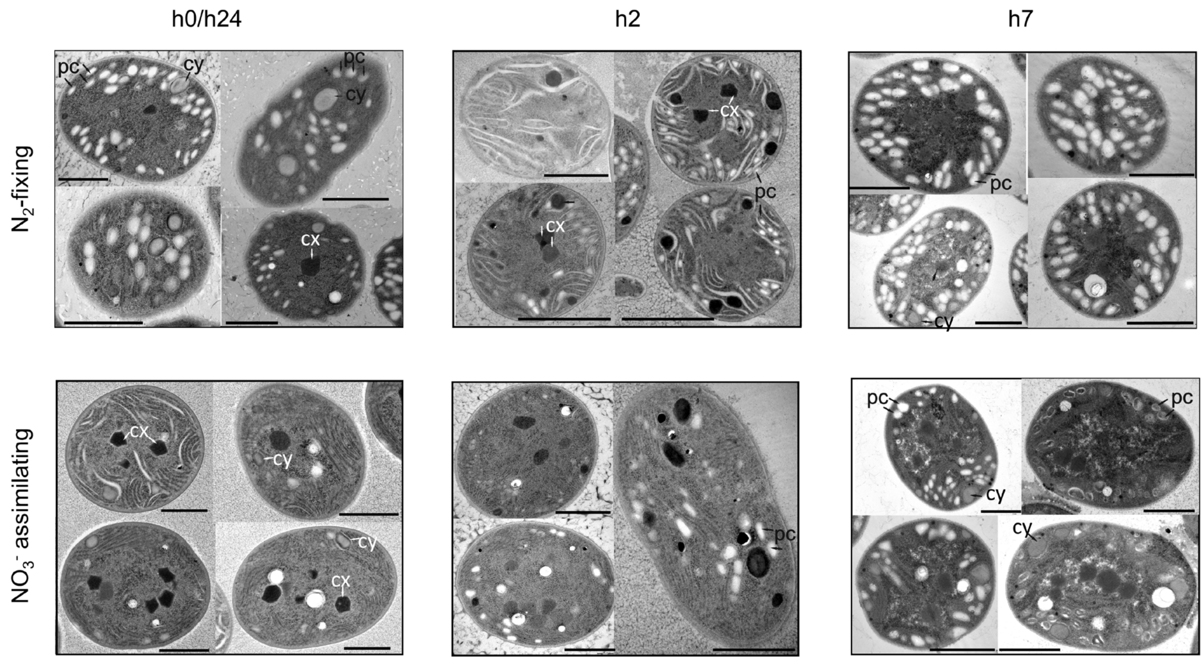

Fig. S1 Additional Transmission electron microscopic images of *Cyanothece* cells harvested at h0/h24, h2 and h7 in the light period. Top row – N_2-fixing conditions; Bottom row – NO_3^- assimilating conditions. pc; polysaccharide (C storage), cy; cyanophycin (N storage), and cx; carboxysome. Black bars show 1 μm.

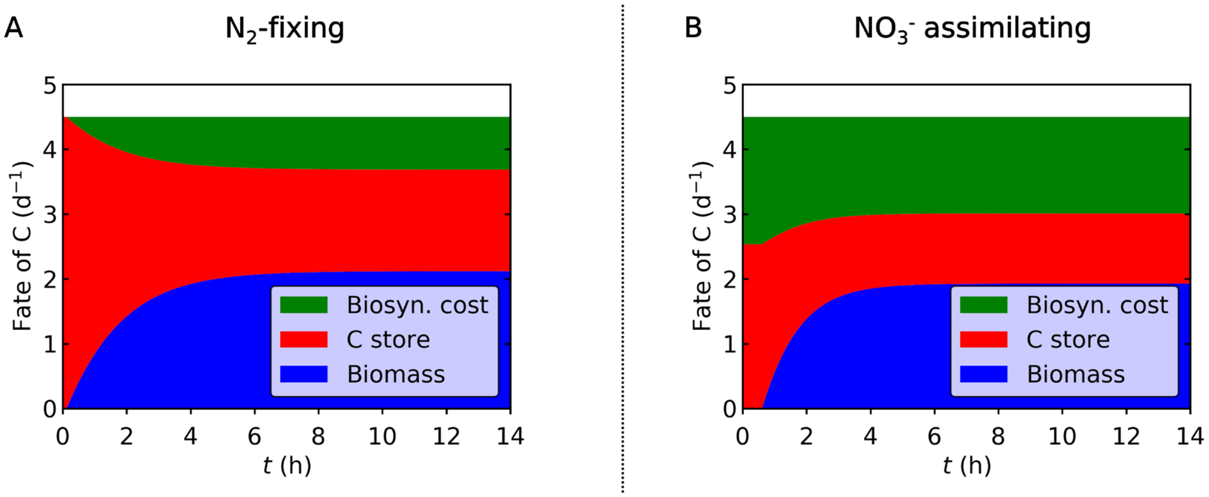

Fig. S2 Fate of newly fixed C during the light period with DIC abundance. (A) N_2-fixing case. (B) NO_3^- assimilating case. Red: C storage. Blue: C for biomass synthesis (growth). Total value represents C fixation rates.
